# Supplementary material for: Antimicrobials as cornerstones and quick fixes Zimbabwean in healthcare and society: Health practitioners´ critical reflections on two stories of antimicrobial use as part of antimicrobial resistance (AMR) education
Source: PLOS Glob Public Health. 2025 Jul 7;5(7):e0004793. doi: 10.1371/journal.pgph.0004793 (PMC12233256; doi:10.1371/journal.pgph.0004793)
Supplement: S1 Data — (PDF) [file pgph.0004793.s001.pdf]

AMR Workshop 2 Harare Group 1

(0:11 - 0:33)
So we'll have 15 minutes about now to share within the group a response and comment on what you wrote. So we'll have 15 minutes to discuss a little, and then we'll go back and look at common themes after that. We'll start with this chair within the group, what you wrote.

(0:33 - 0:36)
Thank you. Thank you. Yes.

(0:36 - 0:45)
So let's have our discussion, and we'll begin by those questions. One, two, three, four. OK.

(0:46 - 0:55)
Whatever you might have not changed a bit, we welcome you. Fine. Get to the tune.

(0:56 - 1:04)
Yes, we'll start with you. I always use the TV. Did you have any new connections? I can start myself.

(1:05 - 1:26)
This for me, you know, yes. I just had some conversations and discussions, you know, with these clients, with these patients, OK, concerning especially the antibiotic use or the injections. Some would say, I'm not good at tolerating orals.

(1:27 - 1:45)
I just need an injection. And some would simply be requesting, I have a fever, but taking the temperature, they were apneic. So you simply say, fine, we'll try to treat you conservatively without any antibiotic or just give you some paracetamol.

(1:45 - 2:02)
But to some, it didn't work well, especially this with flu. So they wouldn't agree, especially for the conservative treatment. And we would have others who would come in, can you write me a prescription? I want to keep acitromycin.

(2:02 - 2:15)
I want to keep blah, blah, blah, blah, and so forth. So these were some conversations which I had with the pre-papa. But this time, I was not a bit cautious.

(2:15 - 2:29)
Yes, but however, the surgery, well, we spend that day looking for money. Sometimes you want to retain your clients, and sometimes you give in to their demands. Yeah, so you always strike a balance.

(2:29 - 2:42)
You want money, you also want to educate them on antimicrobial resistance. Strike a balance. By the end of the day, that's over to you.

(2:45 - 3:11)

So did you have new connections? The new connections that I had, you know, even until now, we hadn't talked about the use of antibiotics. Because usually, we are talking of ANC visits, early bookings, especially the ANC. The antibiotics, we only talk about them usually post-delivery.

(3:12 - 3:39)
So, yeah, I did have a few new clients. The community around me, the clients that are coming in for ANC visits. Then the consultation that we had, we talked about, you know, the compliance that you mentioned earlier.

(3:39 - 4:08)
And the conservative treatment that you also alluded to. Yeah, with the type of community that we have, and the type of clients that we are having, they would have tried all other things before they even come to you to seek for medical attention. So if you talk about AMOXO, it's the first line of treatment.

(4:08 - 4:21)
They would say, ah, AMOXO doesn't work for us. I tried it, you know. So they are coming to you to seek for these other drugs that they cannot access in the industry.

(4:22 - 4:46)
Or, you know, the way in which they cannot buy without a prescription from the pharmacist. So it had a lot of impact because they are doing it unknowingly. Not knowing the implications of using these antibiotics just to explore.

(4:47 - 5:12)
Is this new drug that is coming on the market now, is it? But is it really indicated for your condition? Is it the first line of treatment that you will do? That's what we talked about. Though they are still reluctant to say, ah, you know, I'm very comfortable with the injection. Because with the oral drugs, I might forget.

(5:13 - 5:20)
I really forget. So I would rather have my injection. This is the conundrum that we are having.

(5:20 - 5:41)
And even among ourselves, they would say, I need to wait from 7 to 7. And usually maybe I become so busy that I can't think of my medication. By the time I think of my medication, I will have skipped maybe. Depending on the type of antibiotic that they are taking.

(5:41 - 6:04)
I might have skipped two oral antibiotics. Which means the compliance there is compromised. True.

Yes, yes, yes. This is the first question that you answered. Yes, you were tempted by that, did you? Okay, so I wasn't there but I was given a feedback.

(6:05 - 6:28)

And the conversation was we should use the antibiotics correctly. And so when I was moving around, doing my rounds, I was educating the cleaners not to mix disinfectants with detergents. And also to make sure they first clean, remove any spillages.

(6:28 - 6:38)
And clean with water. Then disinfect the area. Then later on remove the disinfectant.

(6:38 - 6:59)
And also not to use disinfectants always when we are cleaning in the wards. That's what I did basically. And we also did the training on... And also on the unnecessary use of injections.

(7:00 - 7:13)
You remember the workshop that we did. We did the advantages and disadvantages of not giving injections unnecessarily. Where we said we are giving oral saves time.

(7:13 - 7:35)
We don't need to dilute and it's less expensive to the patients. Though we discussed that you want men as private practitioners, you cannot do away with antibiotics. And also the problem that we noted as a central hospital is that these patients, when they come here, they have already received the antibiotics.

(7:36 - 7:42)
And they are very sick. They cannot swallow the tablets. So sometimes we cannot do away with injections.

(7:43 - 7:55)
And also another problem that is causing the resistance is shortage of drugs. Erratic supply. Today I'm here, I got in Rocephine.

(7:55 - 8:01)
Then in the afternoon I need the second dose. The drug is out of stock. So I'm going to miss the dose.

(8:01 - 8:10)
You write a prescription, maybe I don't have money. So those are the conversations and discussions that we do. Good.

(8:11 - 8:19)
Right, fine. Yes, I think we have the common thinking. Common experience.

(8:21 - 8:47)
And, you know, common knowledge. Then what was the topic or content of your interactions? This time when I was interacting with my patients, the message I wanted to put through was that not every ailment, not every sickness requires an antibiotic treatment. That was the message, that was the topic.

(8:47 - 9:17)
Not every ailment requires antibiotic treatment, whether oral or intravenous. Okay? And again, there are some people, some clients, some patients who might simply come for the

sake of keeping these antibiotics. Again, there was a care, you know, the topic discussing the care or the use, the careful use of these antibiotics.

(9:17 - 9:39)
Yes, you want to keep these antibiotics because we all know azithro treats that, amoxilin treats that, but it should be used wisely. So people need to be educated and guided. But otherwise the take-home message was not every ailment requires an antibiotic treatment and so forth.

(9:39 - 9:52)
That was the content of the interactions which I did at the staff clinic or even in my private practice. I talked about compliance. Okay.

(9:53 - 10:10)
Because you may notice that a lot of these resistance are coming about not complying with the antibiotic. I'm giving the azithro, I just take it one day and I feel better, then I give the rest. Yes.

(10:10 - 10:39)
Then the next time I get that same illness, when I'm given that same drug, it means no more. I talked about compliance and then I talked about not sharing these antibiotics. I'm given my course to say this is a serious illness, but I find my colleague with the same ailment with me.

(10:39 - 11:15)
Then I say we can share, but I'm feeling much better that we can share. Then I'm also extending the resistance to this one because she's not feeling a finishing of the course. Then to desist from using the street antibiotics because at times the storage of these antibiotics there in the street, some doesn't need light, they need cold temperatures.

(11:16 - 11:34)
Those that are in the streets are exposed to light. Thereby, when you're ever being taken by a client, it doesn't give the results. That person might end up getting another, a second life.

(11:36 - 11:47)
Yet this person could have been treated well with the drugs that have been told well. This is what I had. That was my case.

(11:49 - 12:04)
Content. Then the need for investigations. You don't just need to take the drugs just because you know I'm also treating this.

(12:04 - 12:20)
But you need to hear the blood culture maybe to see if it's still sensitive to you. She took my point though. I also discussed about compliance on drugs.

(12:21 - 12:35)

We noted that health workers are the most difficult people to deal with. Because if I come here with my child, you give me Amoxo. I'm going to ask from my colleague to give my child Rocephine.

(12:36 - 12:48)
Also, there is a problem with pharmacists. They are now prescribing and giving injections themselves. Even if we say, as health care workers at the hospital, we are not going to give this.

(12:48 - 13:06)
If I just go to a pharmacist without a prescription, they can give you because they want money. These health care workers are very difficult in completing their courses. Despite other patients.

(13:06 - 13:16)
Some patients, they fear. They want to take and complete the courses. But health care workers, if I come with the flu, I take it for two days, then I'm done with it.

(13:17 - 13:39)
Then we also discussed on the care of the expired drugs. How are we caring for those drugs? Are they not the ones which are being taken and being resold in the streets because of poor disposal? And for those that we are using at home, where are we putting them? We are just throwing them. Someone can pick it.

(13:40 - 14:03)
That's when they ended up being used to feed our kids. And again, whilst you are still on the disposal of expired drugs, I saw a patient who was in an asthmatic attack. Only to find that the patient had an inhaler which had expired two years back.

(14:05 - 14:20)
That inhaler was sold by a pharmacist. He took those expired drugs and resold them. So there is a need for proper disposal of expired drugs.

(14:20 - 14:40)
If you just send them to the incinerator, someone is going to pick them up and sell them. As for parasites, there will be some tablets in the searches. You can just peel them off, put them in a packet, label it, then resell it.

(14:41 - 14:57)
This is what is happening. Rui, how were you able to incorporate the discussions from the workshop into your practice? It's almost the same thing. Just for patients.

(15:00 - 15:12)
Just for patients to make sure that they get their drugs on time. And they are completing their courses. Yes, and limiting yourself on the administering of IV antibiotics.

(15:19 - 15:34)
And proper adherence. Proper adherence and compliance. Sometimes I find this very interesting.

(15:34 - 15:47)
We have an admitted patient. These days we don't have Rosafil and some of the IV antibiotics. We have this and relatives are asking to go and buy.

(15:47 - 15:57)
The patient is discharged. Whilst there are still some leftovers of these drugs. They know that this was used to treat them.

(15:58 - 16:07)
They keep this anymore. Then when you say, I think you need this antibiotic. The moment you mention that name, I need to give you Rosafil.

(16:08 - 16:19)
Then they will simply say, I have it. Because once they go to the hospital, they always carry the leftovers of the drugs from the discharged patient. Look at it, sometimes it's already expired.

(16:20 - 16:27)
And it wasn't properly stored. And it was not properly stored. They will insist that give me this to cut costs.

(16:29 - 16:53)
So we need to be very, very careful and educate these people. Especially the health care practitioners. Because they will simply say, I have got my sister who works at Paraniyat.

(16:53 - 17:04)
I will ask her to bring. And you guys, the patient eventually dies. But there is still medication for that patient.

(17:04 - 17:16)
Be it paracetamol or oral. You keep them and you take them home. The health care workers, they are also contributing.

(17:16 - 17:25)
Yes. Good. What's there? We are done.

(17:26 - 17:37)
Did your practice change in any way? Yeah. Not really. Because we are... Mine is an individual.

(17:37 - 17:40)
Yes, an individual. Okay. Now I have the conscience.

(17:41 - 17:53)
What are you saying? Yeah, yeah, yeah. Yeah. So there was a change a bit.

(17:53 - 18:02)
Especially the staff clinic there. Fine, they would understand when you educate them. But out there in the private practice, you always try to strike a balance.

(18:09 - 18:18)
Educating these people. Yeah. Because these people are the same people who are saying amoxicillin, it doesn't work.

(18:18 - 18:24)
Even when you talk of these anti-hypertensives. You say, I give you an allopurinol. No, I had a cough.

(18:25 - 18:31)
Then I give you amilodipine. My legs were swollen. You know, and so forth.

(18:31 - 18:51)
Every drug you mention, they always have an effect to that. You know. So, we're talking about a more education inside, integrated in the practice.

(18:56 - 19:04)
So, yes, education. People have to be educated. That when you say amoxicillin is no longer working.

(19:05 - 19:16)
Then, this is a starting point. You simply say, you have been abusing, you have no ability or resistance for that. Remember, we used to treat this as ITIs with penicillin.

(19:16 - 19:24)
But these days, there is no resistance. And they will simply say, ah, there was no improvement. Until you go on, go on.

(19:24 - 19:32)
Then until you take a specimen, you let it swab, and so forth. So, culture is very, very, very important. Yeah.

(19:33 - 19:47)
But at the moment, it's now sensitive to imipenem. It will be not a problem. Do you agree? Yes.

(19:47 - 20:07)
Yes, because they... You know, there is also a need for the community. I mean, you have to educate the community on where to go when they want to seek proper treatment. You know what they are doing? They go to the pharmacist and tell the pharmacist.

(20:08 - 20:14)
I'm here because of your class. If the pharmacist just leaves here, you can take this here. It's yours.

(20:15 - 20:23)
You don't need to leave. So, it's a bit of a discussion. Come, come closer.

(20:25 - 20:38)
Okay, we are having discussions. Last time, yeah, we are having discussions. We are having discussions on antimicrobial resistance.

(20:39 - 21:00)
And our discussions are being guided by those questions which have been put up there. Then our daily experiences, and so forth. Then the idea is to educate people on the existence of antimicrobial resistance.

(21:00 - 21:24)
The abuse, random use of these antibiotics will eventually give rise to resistance. Did you have any connections, any discussions? Yes, we were discussing with our clients. Sometimes the clients will simply say, I'm having a flu, I'm having a fever, but can you give me azithromycin? They can lead you.

(21:26 - 21:36)
But when you are out there, you need to strike a balance. Yes, you say you can get resistance to that, but at the same time you want money. So, you always strike a balance.

(21:36 - 22:05)
But you need to conscientize the people. Some patients, clients, they want to keep these drugs for themselves, so that when they feel that flu, that cough, they will simply go to their private pharmacy, and so forth. Do you know that we are not only getting antibiotics when we are sick? We are getting antibiotics from the chickens that we are rearing at home, from the fish that we are rearing at home.

(22:05 - 22:22)
Antibiotics are being used today. Some people are using antiretroviral drugs to feed their chickens, so that it becomes so big. So, I might not be going to hospitals seeking these medications, but I'm getting it from the food that I'm eating.

(22:22 - 22:48)
It's no longer natural food. So, I don't know what in general the community likes. I don't know the type of vehicle that we are going to use.

(22:48 - 23:14)
Is it the social media? Is it the church leaders? Is it the traditional healers? But if we start with us, because we are the ones who tell these people that these antibiotics work like this. Yes, I started with us. The community doesn't know.

It's us. I've seen it on Facebook. I've seen it on Facebook.

(23:26 - 23:44)
I've got my aunt at home. When she comes, I make sure I give her as if it were my sin. When I could write for Chakat, I give her Kefloxa for UTI.

(23:54 - 24:07)
Even when the children are going to a boarding school, you give them a packet. Paracetamol, Citro. Yes, I give them Citro.

(24:10 - 24:20)
Omeprazo. So, you give them Citro because it's taken twice a day. The three days they will miss, you give them Citro because it's taken once.

(24:20 - 24:29)
Once a day for compliance. So, we are contributing. We are the contributors.

(24:29 - 24:39)
Yes. There are people who don't want normal drugs or just symptomatic therapy. For example, the flu issue.

(24:40 - 24:48)
If you tell them, you just need supplements and something for the pain. It's a viral infection. They are like, no, I want those antibiotics.

(24:50 - 25:10)
That's why it's a challenge. Yes, okay. I think we... Takeaway message.

(25:14 - 25:34)
I think we've had some time to share our experiences in the practice and also really found some common themes throughout our discussions. So, before we move forward, let's just take a break to refresh ourselves. There is tea outside.

(25:34 - 29:25)
And then we'll come back, let's say, in 10 minutes. So, we can start again. How are you? I see that it's not there anymore, isn't it? You need to clean it.

(29:25 - 29:32)
I don't see it anymore. I don't see it anymore. It's not the same.

(29:33 - 29:40)
You have to clean it. You have to clean it. It's dirty.

(29:40 - 29:46)
You have to clean it. You have to clean it. It's dirty.

(29:53 - 30:01)
Don't clean it. It has DNA. Don't clean it.

(30:01 - 56:59)
You can see it. I share the same name. Salaam! How could you see that? Hey! You believe that? Shri Mataji, Shri Mat Shri Mataji in Hindi Local funds, poverty, erratic supply, poverty Local funds, poverty, erratic supply, poverty Are you able to give health education to all of them? Are you able to give health education to all of them? The question that we touched on initially today is also that our goal is of course to do immunification The question that we touched on initially today is also that our goal is of course to do immunification The challenges between what is happening in the hospital and the challenges for actual community members and patients to

fulfill that The challenges between what is happening in the hospital and the challenges for actual community members and patients to fulfill that Community values and priorities.

Of course most of us want to be healthy. That's a general value that we share But for all of us, our idea of what it is to be healthy can be somewhat different and somewhat shifting Because of course it's based on our experiences in life So the first question is around how could the values and priorities of what is important for the community be included in our education practices Because we talked about how can we now engage with patients and communities But how could their values and priorities be included? How can we account for that? And also a little bit further outside the hospital, what could support communities in converting AMR education that you as medical practitioners and maybe others have shared with them into better outcomes? What could support this kind of efforts for AMR education? So it's kind of what would be kind of a frame or a support structure for also making the most of those AMR education efforts So those are the two questions I want you to reflect on and share in the groups Does it seem clear? Okay, thank you. Then let's move forward with that question How could communities have values and priorities? We can start with you For me, I'm thinking of communities in the rural areas.

Those people, they value chivanu and traditional methods of treating So how are we going to talk about antimicrobial resistance education to people who don't even believe in antibiotics? I don't know So how are we going to support them? How are we going to bring those two together? So that we are still valuing their views and their priorities, but we also want to teach them that taking antibiotics, abusing the use of antibiotics can lead to resistance And I agree with them, especially in my trainings. Like we were saying in my chief years, we don't need a vaccine. We can program any training, we can go out and buy one We need to be able to analyze the community for not following whatever is being instructed to us.

(56:59 - 58:17)
We need to be able to analyze the community for not following whatever is being instructed to us. I don't want to talk about it because the people who were born don't know what antibiotics are But we are saying it's not over yet It's waiting for them But I don't want to talk about it because if you put them, then you can move forward with your goals We should engage the people in power. Those chief people, they have more power.

People can listen to them My mother is sick. She has an outbreak. Is it because the vaccines were not good? No, we don't give MR vaccine.

We are not giving you the MR vaccine. It's measles and rubella. We should have MMR.

(58:17 - 58:25)
But we don't have MMR. But when we started, 9 months ago, you gave MMR. Yes, but practically it's not being given.

(58:26 - 58:33)
All the time, I'm like, they received it. They received it 9 months ago. I didn't know.

(58:33 - 59:17)

We are sort of addicted, not because of this presentation, but we would like to discuss If we are to win those communities so that they adopt the treatment regimes or the education that we want to disseminate to them We need to improve the community of us. And if we want to get to the churches, we need to get to the church leaders so that we get to them. So we will be sharing our expectations and we will be sharing what we believe.

(59:17 - 59:39)
Maybe if I can make it true. Of course, these mainline churches, the Anglican, the Catholic and so forth, within their set up, they also have some communities. They do have health communities.

(59:40 - 59:59)
Sometimes they can educate people during the era of COVID. The COVID pandemic. They would look for customers, educate them, make sure there is water spacing, washing your hands.

(59:59 - 1:00:14)
They would emphasize that. Only for the other indigenous churches where health is still a problem. But otherwise for the communities, they value the essence of maintaining a good health.

(1:00:15 - 1:00:36)
And even their priorities, they know very well that if you fall sick, then you should get treatment from clinic, hospital and so forth. Because others can go to traditional healers, the minority. But they need to be educated to finish the course.

(1:00:37 - 1:00:49)
Because they will simply say, I am also having this problem. No, I have the leftovers and so forth. So their priorities, once you fall sick, you should seek medical attention.

(1:00:50 - 1:01:01)
Completing the dosage is now something else. Compliance is now something else. As long as you have visited the clinic and so forth.

(1:01:01 - 1:01:14)
That's their priorities, that's their values, that you fall sick, go to a clinic or hospital. No one will simply do a follow-up sometimes. We do have even some elderly people.

(1:01:15 - 1:01:45)
No one will supervise whether they are taking their medication consistently or not. And those of them who have the dosage being taken, especially QID, they might not take the medication continuously. Through the committees that they have, like the health committees, they can even make follow-ups to their patients.

(1:01:46 - 1:02:15)
Once they know that the patient is ill, they will have duties to say, today Sorenson is going to see a patient. And even for the elderly, to say, she is taking her medication, she is eating, is she mobilizing, is she communicating, all sorts of things. So, in a positive moment, that's the only way to get rid of it.

(1:02:17 - 1:02:33)
In some communities, they are now making use of the pill boxes. Monday, Tuesday, Sunday, Monday, Tuesday, Wednesday, Thursday, Friday, Saturday. Every morning they put some pills on that in the beginning of each week.

(1:02:33 - 1:03:05)
Then they can monitor whether today is Tuesday, is the Tuesday medication taken, today is Wednesday, did she take the Monday and the Tuesday since today is Wednesday. So there is a way also of monitoring and also making it easier for the one taking the drugs. Sometimes we are taking once in the morning, you know, then the three drugs are put in.

(1:03:05 - 1:03:57)
So they can also make life easier for those people, so they are making use of those pills. I always give an example, you see, you remember that circumcision, which was done by the musicians, the Jack Reza and Isabelle, when they want to sing a song in line with what they want the community to be happy with. I don't know how we can make it in school.

(1:04:00 - 1:04:19)
Yeah, we can also make use of it in schools. I know most of the boarding schools, they do have a clinic there. Most boarding schools, they do have a clinic, they run a clinic.

(1:04:19 - 1:04:41)
So they can also be, education can also take place even in primary, secondary school, you know. And they can even educate their parents at home to say, I've been given antibiotics, I need to finish this. I'm taking it after feeds or before feeds.

(1:04:42 - 1:04:53)
Because science is somehow taken in well. Working with the kids in the schools. Yes.

(1:04:56 - 1:05:05)
How about we should start with the health care workers? Yes. They are the ones who are causing all these things. The health care workers.

(1:05:05 - 1:05:18)
It's true. So I've just seen a patient with side effects of paloperidol. And I was like, we told you to just take 5mg Nocte, but the sister said, no, you should take it twice.

(1:05:18 - 1:05:32)
He was taking 10mg per day and he wasn't taking the Benzoxol for the side effects. Now the side effects are so bad, he's protruding his tongue, he has smart tremors and he's so young. But it's not tolerable at all.

(1:05:35 - 1:05:51)
Nancy, please. This particular one came across, by the time I was still staying in Eiffel Tower. These red prostheses and these even other ancillary stuff which is working here.

(1:05:52 - 1:06:08)

Whenever they hear that someone is working in the hospital, they assume they know. I was working as a paediatric casualty. If you come with your child, Dr. Chimonzo won't just prescribe any medication for you.

(1:06:09 - 1:06:27)
He will start by asking the sister, which medication do you want? Because he knows you have dealt with everything. I'm not denying that, but I can also say, like I was working on the tinner nest for my wife. I was working on the tinner nest for my wife.

(1:06:27 - 1:06:48)
I was working on the tinner nest for my wife, especially when I was working on the kitchen. I was working on the kitchen and the staff were not taking my wife to dinner. But it's over.

(1:06:49 - 1:06:57)
It's over. We should have them on duty. And they also judge them.

(1:06:58 - 1:07:03)
Now, I was a kind of abscess. I got a lot of pain. And it's all day.

(1:07:04 - 1:07:11)
I got injected. So, we should do something. It was a very bad abscess.

(1:07:13 - 1:07:17)
Yeah, it happens. It normally happens. So bad.

(1:07:18 - 1:07:33)
I think they should be nursed. What about the next question? It was like the side effects of some of the drugs. You know what is happening.

(1:07:34 - 1:07:46)
Instead of coming back to the doctor, to the nurse, I am explaining this. They just talk on the phone. When you say, your BP is very high.

(1:07:47 - 1:07:53)
Your medication made me that, that, that. I was so weak and what? You know. But it should come.

(1:07:53 - 1:08:11)
Then we will discuss about the better medication. If you have been given the medication, why do we have to support the communities? Why did he complete the surgery? Support the communities. They did not get a higher education to better their outcomes.

(1:08:11 - 1:08:32)
It was so painful. We are taking it from the history. So many women do not experience without the prescription.

(1:08:35 - 1:08:51)
If you do this, maybe if you provide to your children, you give them an incentive. To stick to the medication. But it is not sustainable.

(1:08:52 - 1:09:05)
If you give an incentive to say, come for education. We want to educate them. But for us to... To bring them to the health facility.

(1:09:06 - 1:09:15)
Maybe to incentivize them to say, everyone is coming, give them $10. Maybe. Maybe just for the health.

(1:09:17 - 1:09:33)
People will come for anything. There are mothers who are coming here at Central Hospital. They have breastfed their children for two years.

(1:09:33 - 1:09:46)
They were told to breastfeed for two years, then you get a bicycle. But those things don't exist. So when they go back to the local clinic, looking for their bicycles, they are referred to Salim Kabisa.

(1:09:51 - 1:10:07)
There were a few bicycles, which were given Metro and Benzene, acknowledged to that. I don't know, but she said yes, it is the program, but extended. People should know that breast milk is the best for the baby.

(1:10:08 - 1:10:21)
People are coming from as far as Aruba, to come and get bicycles, when he doesn't even know. My child is just 22 years today, and I'm about to be an adult. I've come for the bicycle.

(1:10:22 - 1:10:34)
For the reward. You know, there were certain things, which were done previously. But, a lot of things have changed.

(1:10:36 - 1:11:00)
We used to, at the nursery, they specialize in the treatment of TB. Once you are diagnosed with TB, you come every day, you get your medication, then you go to the hospital. Then you take your medication, in their presence, when you come.

(1:11:00 - 1:11:13)
If it is for six months, you come every day. If you fail to turn up, look at your address, they will do it for you. They even screen your family.

(1:11:14 - 1:11:27)
But these days, they will simply say, this is three months, or you take your medication at home. The population has grown. The town, this is not the Harare that you used to know long back.

(1:11:27 - 1:11:45)
And people, you can tell them, they come every day, they can't afford getting transport every day to come. Yes, where it's a walking distance. So this is what is happening.

(1:11:46 - 1:12:08)

And sometimes I do teach them, that all the doctors used to treat, you know, at present, we don't treat you, but we are assisting you in the management of your own health. I give you a prescription, you don't come to me and say, I am going to take the medication. So if you don't buy, it's your own look out.

(1:12:10 - 1:12:39)
If you buy and you don't finish the dosage, the medication, I will change you. I will go to you. So try to drive home and say, gentlemen, if you don't take your medication religiously, A, B, C, D, W. And if you find this company, drug resistance, you know, and especially the HIV, quite a good number of them have put them on the second line.

(1:12:40 - 1:12:50)
They were defaulting. And what happened, at first, like the first one, at first, the viral load was 2000. Then it defaulted.

(1:12:50 - 1:13:05)
Come, defaulted for three months or so. When it came, you know, viral load was already 221,000. But if you are talking about all my patients, those patients that get HIV cancelling.

(1:13:06 - 1:13:39)
So that man, he said to me, I will never, ever stop taking this medication because otherwise I will die. He saw it by himself, and I said, go and preach to other people. Yeah, you have brought me to the same idea that I had when you said to tell to make it normal to those that have taken up the course and they've recovered, they can act as, you know, to inspire others.

(1:13:39 - 1:14:02)
To say, you guys, look at me, you showed to me, you know, it's a horrible issue right now. But if another woman recovers, to say, you guys, you showed me when I was unwell. But I need to have education and see where I am today.

(1:14:05 - 1:14:20)
And also I was thinking of what it seems I'm quite passionate. Can we play it? No. I was thinking of the use of dramas.

(1:14:21 - 1:14:42)
You can use a drama with a negative picture. To say, what happens when you don't take your antibiotics religiously. And what happens as well when you take the two sides being good.

(1:14:44 - 1:15:01)
Do you have those stories? Do you know the soldier game which was used in HIV? The soldier game is the MRCD for Corona. A lot of things used to be done before. I'm fortunate that these days.

(1:15:02 - 1:16:24)
You know, when they established the Masters of Public Health program, people said, the medical practitioners, the doctors, you are going to run out of clients because the message which is by those doing Masters in Public Health, they say prevention is better than cure. So

these people specializing in awareness and so forth. At the end, doctors, we are not going to have enough clients.

That was very beautiful. At first, it was okay, but later on it went and so on. So how do we work with the prevention side? So we are now coming back to educating the people on the prevention life and also the effects of these drugs.

Yes, people are going to see a doctor but there is no resistance. There is no education on the dangers of not taking the medication and so on. Because I think they tried using the preventive method but it hasn't taken off.

(1:16:25 - 1:17:06)
We didn't achieve our objective. So we now have that animal in the house the resistance now. Isn't that linked to the question of you could say converting that awareness or AMR education processes? What was hindering that success? So they thought Yeah, but now, you know, we used to every village, health workers village, what about funding? There is no longer enough funding.

(1:17:07 - 1:17:19)
Funding and the economic hardships make people you know, to find other means. Poverty. Yeah, poverty.

(1:17:27 - 1:18:07)
Substance No, what is causing bacteria, we need to prevent by encouraging vaccination. Immunity, they get less sick. Yes, they get those vaccines.

(1:18:13 - 1:19:11)
You are giving this such as, you are giving that but it's all for use of my antibiotics because I have a sister of mine she had a caesarean section in the UK and she was given Amoxy for like 2-3 days. I'm like, here. So what we've done now is that you have time now to come collectively together to reflect on your AMR practice and both appropriately student and for AMR education.

But just to kind of finalize this workshop session, we have about 40 minutes left. So I just want to leave you with another stimulation around these questions. And this is coming out of the research I've been doing and also Dr. Simbini has been doing.

(1:19:12 - 1:19:34)
A lot of these ideas originated in my work in Latin America, in Peru and Ecuador about 10 years ago. I don't know when I was starting out in working with antimicrobial systems more focused. So I call it two stories of antimicrobials in healthcare society.

(1:19:35 - 1:20:05)
And again these are kind of I really like to work with models like illustrations. So I'll share that with you now. And it's not that you necessarily need to agree with it.

It's again a stimulation. So the whole idea is to kind of encourage discussion. So when in your discussions if you see you have another opinion or you see it in a different light, just keep on discussing.

(1:20:06 - 1:20:19)
That's completely fine. No requirement to agree with these kind of ideas. But it's just something to stimulate discussion and throw in what has come out of the work I've been doing.

(1:20:21 - 1:20:49)
So this is quite a common model that you encounter at least around antimicrobial systems in Sweden where I'm from. The first time I encountered this model was from a presentation of a professor emeritus Otto Karls. He's worked within AMR for at least 25-30 years.

(1:20:49 - 1:21:17)
He worked for the WHO and he developed this kind of model that he says that antimicrobials are cornerstones of modern medicine. That's his argument. And he usually puts the antimicrobials at the bottom and he says these are enabling on a first level wound infections to treat wound infections for our able to especially severe wound infections.

(1:21:17 - 1:21:49)
Of course blood infections, sepsis and so on is of course a very big concern if you don't have antimicrobials of course. He also highlights of course urinary tract infections and not to forget pneumonia. Pneumonia has come up in at least the last workshop's discussions and it's a serious report but not antimicrobials, pneumonia is very serious.

(1:21:49 - 1:22:23)
So that's on the first level he presents. The second level here you have for example STIs, malaria on one hand but you also have around like childbirth so you have preterm babies and also complicated deliveries is two of the examples he highlights. Then on the next level he usually highlights organ transplants that usually you need antimicrobials to do organ transplants.

(1:22:24 - 1:22:56)
Also he highlights cancer treatment which is often at least among the general public it's not necessarily always associated with antimicrobials. That's what he highlights there. And ultimately he puts at the top I've seen a few iterations of this but often surgery ends up at the top as a very medical practice that's very dependent on antimicrobials.

(1:22:56 - 1:23:49)
So he kind of creates this pyramid you could say. So I just wanted to start with allowing you in your groups just for like five minutes just discuss your initial impressions of this model. Do you recognize it? And do you think it is interesting? Do you think it would be helpful to use as an education material when you engage in AMR education? And if so, who would you present it to? Would you share it with patients? Communities? Colleagues? So have a conversation first about is this useful? What are the initial reflections around it? And then we'll take it from there.

(1:24:05 - 1:24:29)
My condition you cannot do without antibiotics. When we're doing with antibiotics it's not all antibiotics. Yes.

(1:24:32 - 1:25:38)

The pyramid the pyramid when you talk of a pyramid majority as you go up number increases. The highest number of people who need antibiotics they've got wound infection, urinary tract and pneumonia. Then we go up not everywhere then we go up organ transplants then to surgery Pneumonia because it's not so complicated and the complication I know it's worse but I don't know what it's called in the lands.

(1:25:38 - 1:35:40)
Pneumonia it's called infusion and above it is empyema There are also other positions that may complicate it it's very complicated complications I'm going to follow under pneumonia because it's not the lead of pneumonia Pneumonia is a result of the complication of the upper respiratory tract infection But I was thinking some of the things you just talked about Do you see a use for this kind of work? In a developed country I care about the preventative side because most people they don't have my questions and I'm going to answer Is this something you really like? I don't know what it's called but it's called thyroidectomy and it's an antibiotic Thyroidectomy you need that antibiotic whether it is a clean wound or what normally happens in this country sometimes they forgot to give you and these are the health medicines it was an option it was an option So the wound infections because these wound infections are the patient they know vital infection and also grangriness So I think it could be controlled if people practice healthy they go to the clinics for their wound dressings I don't know This can be controlled if people practice good hygiene perineal hygiene it can be controlled bathrooms are clean things like that but can I wear an eye if I have got urinary tract infection you need to give me an antibiotic yes but also change also change the wound because once you give antibiotic it will go away it will go away and you can't wear it you can't wear it it's true I am resistant to the antibiotics I was saying we need to use antibiotics but we don't have it we have to use not just give when you have someone with UTI you say ok you get it now but you need to change your practice also so you don't get in a gap because UTIs is often due to high deep practices so you need to change something in your practice and if you look at the model as a whole of course Utakashi is a professor, Utakashi uses this as an education model do you see, for example in your own practice do you hear use of this model and if so and with whom would you share it and how would you talk about it if you see a value of using this model in your own AMR education practice if at all you are going to adopt this we discuss it with the colleagues and my colleagues are nurses then the doctors as well we must work as a complement complementation but not with patients we'll find when to maybe when administration or whenever the patient comes in isn't it that in the first discussion we say every young care practitioner should talk about this antimicrobial at every entry point be it the nurse who will serve the patient be it the doctor who will consult the patient be it the pharmacist that will dispense the medication so the patient is our home is our target isn't it so discussing it maybe reaching a certain level that we have agreed and talk the same language then that's when we can go to our patient because as long as the doctor is saying this and me as a nurse I'm saying this there's conflict so then the patient might end up being confused not to know what to follow so we need to talk among ourselves and make a decision every time and sometimes when we give to patients there are some wounds breast ulcer which might be cancerous in the community they also make use of these concussions they don't know whether this is a cancerous wound or whatever but they continue using there are some concussions they use and making things worse so they need to be educated that you can take a password send to hospital take a password so that you come up with the idea of

antibiotic one thing I have noticed some of these healers the traditional healers even the professors and so on they say go to the hospital get your diagnosis then they give whatever they want then they say go back get tested again just to prove that they are also effective that's what I like to find I was thinking about sharing that model with patients sometimes when you give too much information to patients they get confused or they can even start manipulating and they'll be like I have a UTI infection, give me another no, I have gonorrhea as well, give me another so I think we need to be very wise to some extent not everything not everything, yes and you also need to understand the state of the patient you might start lecturing to me this is what I was hearing in denial of the condition also in pain so that we introduce antibiotics plus the old education taking it into consideration the state of the patient on the UTI we treat both physiology on the treatment of UTI we use a holistic approach we also treat the pattern because it might have been already exposed but symptoms haven't developed especially when you are treating a pregnant woman who has UTI make sure you also treat the pattern because two months down the line she's going to come back most people they are afraid of STI they start thinking it's HIV or we are dying they can even start having a fight in the office oh, you are a fighter you definitely remind me of it even if it's necessary a patient should have the all positive not the negative but because I do not want to have someone who says I don't want to have a woman who says I don't want to have a woman I don't want to have a woman I don't want a woman now I want to show But my background is within the global model. I also was thinking, when I saw these models the first time, that when we came, that's all well and good. But isn't it also a little bit of a challenge when we built, if we just take this as face value, isn't it a challenge that we built the whole health care system around one resource? Was my thinking done? So then I, together with my colleagues, developed this a little bit contrary model.

(1:35:41 - 1:36:27)
And this is really the idea that health care protocols, not by its essence or nature, but often in its use, is used as a quick fix for such things as, for example, poor housing. The living conditions of people often is, if people are living in poor living conditions, they're more likely to be exposed to risk of some infection, which often, the really serious consequence of that is kind of a quick fix to antimicrobials. Someone gets so sick, they need to go to the hospital, and they get some antimicrobials and antibiotics, they can go home.

(1:36:27 - 1:36:43)
And that's that. We don't need to fix poor housing. Similar with infrastructure, if you look at the modern sanitation system, we can kind of keep on sometimes running a so-so water system and sanitation system.

(1:36:44 - 1:37:17)
And I've been working in Eastern Cape in South Africa, and the water treatment facilities in many parts of that region is very, very poor, which means that a lot of the times, people are getting E. coli, infections. You also have the infections up in Hammelskraut, in Friesteig, and in the whole thing by the dam. Those things are kind of quick fixed through the distribution of antibiotics and antimicrobials.

(1:37:17 - 1:37:34)

We also have, of course, insufficient waste management, and dumping and so on, and going into waterways where people use that water. We quick fix it sometimes with antimicrobials. Of course, limited access to clean water is quite obvious.

(1:37:35 - 1:37:54)
You can also see, again, with the Hammelskraut example, the political mismanagement of that kind of outbreak of cholera, which is still a concern. You can go into looking at the effective health policies. It's also kind of quick fixed.

(1:37:54 - 1:38:18)
You can argue. This unsustainable productivity comes from a colleague of mine who worked a lot in India, in industries there. And the problem there was that the people who were running the factories were conscious that this was an environment where the workers were likely to get an infection.

(1:38:19 - 1:38:40)
So they were giving the workers at the plant, at the factory, prophylaxis antibiotics, so they wouldn't go, so that they wouldn't have sickness. Because they were just accepting that this was so poor for working conditions, so let's just prevent giving prophylaxis. That's another way of quick fixing.

(1:38:41 - 1:38:57)
And, of course, inaccessible patient care. This group was mentioning that a lot of the challenge of it is that patients come too late. They could have, for example, had, they might have a wound, they could have been dressing that wound, cleaning it.

(1:38:57 - 1:39:25)
It would never have necessitated that the microbes. Of course, lack of hygiene is an ongoing challenge. And I was talking to colleagues at the Padarani Gatwa yesterday and they said that it's quite interesting that at one point they began to reuse the gloves in the hospital.

(1:39:26 - 1:39:48)
And that was, of course, a change. It's, to a degree, a compromise in hygiene rules and regulations, but it happened due to, of course, body thoughts. But that's kind of also those kind of, it's just one example of this kind of lack of hygiene, that microbes can be a quick fix instead of us being forced to solve this.

(1:39:48 - 1:40:23)
We, in the worst cases, we just use them to make remedies. And what I'm writing is that ultimately, this is a quick fix of social inequality, that people, we can keep on running societies where we have a lot of inequality and the really lethal consequences of that inequality is kind of coming over or quick fix to that microbial use. So that's one way of seeing it that I just wanted to present to you.

(1:40:24 - 1:40:41)
And I can preface this by saying I don't think these things happen at the same time. It's both a cornerstone and an important resource. At the same time, it is something that is often used in a way for quick fixing other social challenges.

(1:40:42 - 1:40:54)
So it's this kind of, the antimicrobials are neither good or bad. They are biotechnical innovations, you can say. But how we use them is quite an interesting point.

(1:40:54 - 1:41:26)
So I wanted to just encourage you to have a moment to discuss this idea I'm throwing out now to you. And you don't need to, as I said, agree with it necessarily, but it's a kind of a stimulus for discussion that I wanted to share. So the first question, I guess, is your intuitive thoughts about this second model and its relation to the first.

(1:41:27 - 1:41:56)
And the second question is really how can this, could this be useful in antimicrobial resistance education? And in that case, in whom and how could it be useful? I don't see that. They work together, yes. Because these are the real issues.

(1:42:01 - 1:42:15)
Yeah, so that's a paradox. So they're saying that the real issues could be these ones which are leading to the infection. That will lead people to take the antimicrobials, which is actually true.

(1:42:16 - 1:42:38)
But at the same time, we also need those antimicrobials in the event that someone can have all these things like the normal structure, normal housing, but still get an infection, then we still need the antimicrobials. True, yeah. And I also, when I say the quick fix, I think these are the risk factors.

(1:42:40 - 1:42:54)
These are the risk factors. I have got poor housing, you are exposed to TB, crumbling infrastructure, poor clinics and so forth. So those are the very risk factors.

(1:42:54 - 1:43:02)
That causes the infections. That causes the infections and the need for the antibiotics. We could fix that.

(1:43:03 - 1:43:17)
Then, you know, if you have got limited access to clean water, you are exposed to typhoid, cholera. Now and again we experience cholera. No reliable tap water and so on.

(1:43:18 - 1:43:34)
Insufficient waste management. Go to Mbarebu. You know, we don't have... Go to Mbarebu, Sika, or even within our communities, waste is just thrown everywhere.

(1:43:35 - 1:43:41)
Anywhere. For dumping waste. And so forth.

(1:43:41 - 1:43:57)
All the communities around that dumping site. I think we need to engage the people, the policy makers, the counsellors, the people who are actually in power to make changes. The people who build the water structures.

(1:43:58 - 1:44:09)
Those people who make decisions where we need to build this here. Those are the people who actually drive the idea where there is poor housing in these areas. We need to build houses.

(1:44:09 - 1:44:24)
We can raise these issues to the people who are policy makers. I don't know, the people in politics. People in politics are interested in retaining power.

(1:44:25 - 1:44:37)
Their position. Consolidating their position. Do you think they don't know that there is an emerging European power? Do you know? But they don't want it to be said.

(1:44:39 - 1:44:46)
Give me a comment. You know, I was on duty on Monday, it was a week. I was on duty.

(1:44:47 - 1:46:05)
Then they told me they won't turn it around. When the general again was trying to push to say that there is an emerging European power, they told me there is an emerging European power. I was on duty on Monday, I was on duty on Monday, I was on duty on Monday, I was on duty on Monday, I was on duty on Monday, I was on duty on Monday, I was on duty We definitely need some people, but really, I don't know, I don't know what to say.

(1:46:05 - 1:46:13)
In the first year, the policy is unsustainable productivity. Unsustainable. That is so good.

(1:46:13 - 1:46:40)
All right. Thank you so much. Thank you so much.

(1:46:40 - 1:46:47)
So those ones, ineffective health policies. Inaccessible. Inaccessible patient care.

(1:46:47 - 1:47:22)
Inaccessible patient care. Lacking hygiene. So how could we use it? If you're thinking about the AMR education, you touched on it.

(1:47:23 - 1:47:45)
Who would you, if you were using it? The policy makers. Then the policy makers, they will then send the information to my head of department, and to my head of constitution. As long as they are consulting, then we implement.

(1:47:47 - 1:48:06)
The political mismanagement of epidemics, you know. We recently experienced the COVID pandemic. Money was given, you know, for that money.

(1:48:06 - 1:48:15)
They would simply say, gloves. It was in the channel, the way it is supposed to be. They would ask you to use the epidemics to save it.

(1:48:15 - 1:48:32)

You remember the former Obdaya Moyo. Gloves which was costing $3.50, $60, $50, and so forth. Those, they should be channeled where they are needed.

(1:48:34 - 1:48:43)
You know. Even if when, ineffective health policies, there should be a follow-up on whatever is done. There should be a follow-up.

(1:48:43 - 1:48:51)
Sometimes there is no follow-up. Insufficient waste management, do you know? Yes, I can't. We don't even have working scenarios.

(1:48:53 - 1:49:01)
I was shocked to come to the people of Odozi. We don't even have one in the whole country. There would be some.

(1:49:02 - 1:49:22)
Waste management. In Odozi. So, we can say my policemakers, they can make policies, but they need to be educated first, my policemakers.

(1:49:22 - 1:49:32)
You can have very good policemakers. Implementation. By the end of the day, those policies should be implemented.

(1:49:34 - 1:49:42)
Not just on paper. Get into an office, these are the policies, that, that, that, that. But on the ground, are they being implemented? So, there should be a follow-up.

(1:49:43 - 1:50:00)
It's not just a matter of saying, we laid down these policies. Who is going to do the follow-up? Because they should be channeled down through the main industry. Unsustainable productivity, hunger, poverty.

(1:50:00 - 1:50:13)
You know, most of our people are subsistence farmers. If there is sustainable productivity, they can avoid these things. Some of the things that limit access to clean water.

(1:50:14 - 1:50:27)
You know, we can do away with bilateria, diarrheal diseases, water-borne diseases. We are just wasting resources. And then, if we do this, the prevention cuts.

(1:50:27 - 1:50:53)
Most of the things that we wish, we wish to manage better. Very good. Why? But it is on scale.

(1:50:57 - 1:51:45)
It is on scale that the sickness cuts. Waste management, inaccessible patient care, inequality. The rich-poor gap is increasing our people.

(1:51:55 - 1:52:09)

She is like a tiger, like a lioness. Right, what else? Crumbling, crumbling. That should be crumbling.

(1:52:09 - 1:52:23)
Crumbling. Yes. Because we used to have villages also.

(1:52:24 - 1:52:32)
My family used to have a village. My family used to have a village. Common peels, paracetamol, amoxicillin.

(1:52:32 - 1:52:37)
We used to treat our sick kids. And so forth. And they were giving disinfecting condoms.

(1:52:42 - 1:52:54)
And if you talk about the growing population, there are no new residents. There are no camps. There are no schools.

(1:52:55 - 1:53:06)
There is no water. So the town planners are not there anymore. Even the town planners, even the rural council planners.

(1:53:21 - 1:53:30)
I am sorry. She is not sure what to do. She is not sure what to do.

(1:53:34 - 1:53:40)
I don't know what to do. I don't know what to do. What do you know? I don't know.

(1:53:43 - 1:54:04)
I don't know what to do. But anyway, the next one. Not a way.

Not a way. For it to be successful. I can't wait to get blood culture.

(1:54:05 - 1:54:35)
I can't wait to get blood culture. So, in a boring way, I did your research, blood culture, necessary data, and I did your research, and I got these two MCs, I just got the blood culture data, my resources, I got the blood culture data. Are you able to regulate the age of the people you're teaching? Yes, but I don't, I'm not able to, I'm not able to say, hey, how are you, what do you want from school? I'm going to say some common things, basically.

(1:54:35 - 1:54:44)
I have some names now, you want to distinguish? Yes, I want to distinguish. Most of the time, we actually bought it daily, and most of the time, we don't know the pieces of it. It will go away one day.

(1:54:45 - 1:54:55)
How? It will go away, two MCs. Then, upon producing evidence, that way, it doesn't go away. But it will go away one day.

(1:54:56 - 1:55:02)
And how soon? And how soon, I don't know. So, I'm saying it's unpopular. It's unpopular.

(1:55:02 - 1:55:08)
It's in the centre of the clinic, it must be there. It's in the carousel. It's in the centre.

(1:55:08 - 1:55:21)
We have no window for it. No window for it, so it's over there. I mean, so it's the risk of wanting to have the blood culture data to confirm it.

(1:55:41 - 2:21:50)
I think we have touched it a lot, a little bit, we have touched it a lot, okay? Sustainable productivity, food, food production, food security, we are talking of food security Because you can also link it to, for example, overuse of antimicrobials in the rearing of cattle and chickens You make them, you combine them in a small space and naturally they will be getting sick But you give them prophylactic antibiotics and antimicrobials and then they will still be able to live in that confined space So it's both the work conditions but also of course the food production Besides them being sick, we are also taking those antibiotics from these animals that we keep for our own consumption Like they are saying, there was this issue of A.R.V. being given to chickens Maybe when I eat that chicken which is A.R.V. Suppose now I get A.R.V. infection So reading up on the withdrawal time for meat And the recombination is between 80 and 100 days after antimicrobial use for slaughter Which is quite a long time, this is 3 months If you save the chickens, I have to say that it's 6 weeks 6 weeks, they should be mating Then it becomes antibiotics Okay everyone, thank you so much for your discussions I think our time should be just arriving I just wanted to bookend this workshop, these two workshops So we can just have the two groups, you've been working again separately Just share some major themes or takeaways you got out of this session And also maybe this workshop and the previous workshop So we can just have some sharing before we conclude our session So maybe we can start with this group What were your major themes or takeaways from today? Connections, conversations There were some guiding questions New connections, new conversations No need for you to think The first one says, do you have the new connections, Conversations or discussions after the previous workshop? Our answer is yes, we did have new connections That is, with our colleagues Finally we would talk before, but now we were talking using the information that we had acquired from the workshop Trying to make H1C, the need for you to consider certain concepts before prescribing an antibiotic Then we talked about the conservative treatment Where we would maybe educate our clients to say it's not all about antibiotics We can use some other treatment regimes which doesn't include antibiotics Why are we trying to prevent resistance in us and them? Because whenever you're talking to someone and you talk about them as if you are outside them It becomes something, so you need to include yourself as well To say I'm also susceptible to resistance if I use these antibiotics In the same manner that I'm talking to you Then we also talked about the environment We have assumed that the waste management, the waste is the most causative organism That are predisposing our environment to microbes which would later get to us We talk of water, you find people, most of the day they don't have clean water The only time that they get water, maybe the reservoirs are very small So they will end up getting maybe well water which is not protected Maybe those ones from the river But look at the river now, it is being used as a jumping site by people So we are being exposed Then the environment also, we were talking about We talked about the farming area Farming also includes poetry, isn't it? Or piggery, fishery, we also use antibiotics in those types of farming But then he was also

educating us to say He was doing a research and he was saying After you have administered an antibiotic to your animal or to the chicken You need to wait for three months before you slaughter that Yet we are saying our chicken, we are saying it has to go for six weeks Why? Because we are saying it will consume a lot of feeds By three months it will be like a goat And to me there is no profit so the business collapses So we are exposed through the farming methods that we are using So we also talked about trying to run away from the use of injections unnecessarily Because our community, they feel if you are not given an injection You have not been treated, especially when they come to these quaternary levels If it was at the primary level maybe they would accept it They would say maybe I will go for the secondary level But we are the quaternary and if we don't give injections They would say is it possible for me coming all the way from Rwanda Coming here and I am given an oral antibiotic, no injection So we need also to work on our customs and traditions And our beliefs as well So that we incorporate this AMR education Then we also talked about the unavailability of these antibiotics This day prescribes an antibiotic Maybe our pharmacist doesn't have it The relative goes to buy, they come with a day supply Then the next day the patient doesn't have medication Maybe after two days, three days they manage to buy another dose It also increases the chances of us getting resistance to these antibiotics Then we talked about poverty Poverty, poverty, that's the bottom line Poverty is crippling us To an extent that we are buying those antibiotics from the streets Which are expired or maybe even storage The storage is not the proper storage for the drugs that we are supposed to consume Which is a health hazard to us Then on number two I think some of the things were interlinked So on number two we talked about About the diseases, it's not every disease that requires an antibiotic That was our major And the care of drugs, as I was alluding before The compliance in storage The need for lab investigations before we prescribe any antibiotic Then the pharmacies now, I think there's a need for involving the pharmacies The outside pharmacies Until the hospital pharmacies, some are here But the outside pharmacies, they are also prescribing injectables to patients And even going to an extent of giving those injectables to the patients at the pharmacy level Which is a hazard as well Then the care and disposal of the expired drugs He was alluding to a scenario that he witnessed a patient coming in with an asthmatic attack But the inhaler that he had bought was two years expired But he had bought it recently So maybe our pharmacy disposes the expired drugs there Which is not the normal disposal But there are scavengers who come and scavenge those thrown out drugs Then they go into the streets or into the pharmacies and resell them You know, it's something else that we talked of Then we talked of number three, which said how were you able to incorporate discussion from the workshop into the workplace We talked about youth education on random use of antibiotics And the need to desist from using street medications The administration of drugs to us, I mean us as youth practitioners We were saying we have to adhere If it's an eight hour drug, we should strictly adhere to eight hours Not to say whenever they feel like giving Instead of 1400 hours, they would give it at 1800 hours It increases the susceptibility of one getting resistance to the antibiotics I think that's what we talked about Can we have a look at the other group, what you discussed about? So we also had similar ideas The first one, the first one was the poverty I was actually excited that you also put that up So poverty, you say that it influences our populist response to even our teachings and education on AMR We talked about engaging the policy makers We need to engage them into this AMR education Because we can't do this alone, this is actually bigger than any of us We need those that made those policies to also be sensitized To the fact that if we do not take heed We might in the future

not have any medical drugs to be using actually We may want to discuss and go back to this point To implement AMR in the workshops, we need equipped institutions We need institutions with available drugs, a variety of those drugs We need institutions with the manpower We need institutions with even the cases, the summaries to be using For lab investigations, collections, to be taking samples as well Because we don't want a scenario which may be what we have right now Whereby a patient comes in, they have to buy the culture bottle They go out, purchase the culture bottle, come back, we send them back again We take the sample, they go back out to a private lab to do that test We wait for another 2 weeks for even a preliminary result And the patient is still seated there, maybe with no drugs being administered Or with just Ketraxone, Ketraxone, Ketraxone And having a situation where we say that maybe we can buy just a simple dose for today Tomorrow there is no drug, the next day no drug again And we have yet to be able to test it, that is the reality on the ground So we need more equipped institutions We really wanted somehow to demystify antimicrobial usage To make it more, for our populace to be able to relate with what we are talking about To make it relatable to them So we thought maybe we could use celebrities or give, not incentives as such But maybe a reward system somehow We can give antibiotics to finish your course That equals good health or good recovery Just some positive reward to encourage them to follow protocols And then you say, ah, some protocols ignore clinical pictures And we have protocols that have been set in place For example, the fact that BRIDH organs were not safe to patient Without a sputum positive result So we have these numbers, we've got TB patients in the wards With other patients who are also ill With visitors, with us, the health workers We're having to wait for a couple of times, for the first two weeks And we have the patient housed in the general ward Resistance, it's really an anti-allergy system So where are we going, where are we going We need to also look into our policies, our protocols So that they are not as safe and rigid But they can also accommodate different scenarios that we're facing every day Then we talked about subsidizing health services But we had a very clear request We realized that if we do subsidize our health services It will actually decrease the quality of health services to the elderly people So somehow, we distracted by this We started subsidizing the health services And offering the health services to people So we talked about partnerships, private partnerships We developed the institutions, if possible And then we wanted, on the pyramid, I will discuss I was very interested in practicing on That if we take away the inequality from the top Then everything falls down There's no micro-virus being used as quick fixes Because then the inequality is not there That hierarchy that we had at the top, the last part So if we remove the inequality Then all those inaccessible locations will be left empty They go down So we need to engage policy makers We really need to engage them somehow, some way And to demystify the pharmacological aspect of AIMAR to the public To make it relatable to our people We talked about our values as well So we talked about taking AIMAR to teachers So they can also teach to the children Because in our society, we respect education We respect what the teacher says So if the teacher says, these are now anti-micro-virus They teach on anti-micro-virus They teach even on the resistance They teach on anything The children and even the parents The populace will be sensitized And it will have an impact Because what we're trying to do is spread knowledge People need to know, be well informed Be it at a tertiary level, be it at a primary level If we start doing that Thank you Very good, very interesting inputs So I think it's also, to just finalize here I think there is a point to make And this is really what comes also back To this kind of question Back into the purpose Why are we engaging with AIMAR And I think we've touched on even more

purposes But these are some of the ones that emerged from here We have, this should be spread So we can think about it Traditionally of course we're not in the corner there We think about it as preserving and producing anti-micro-virus But we've also discussed the question of How do we produce the reliance So we're not just putting all our hopes to The pharmaceutical industry to produce new Length lines of antimicrobials But then we come into this question also That we've touched on initially This is the preventive and addressing of emergence of AIMAR Which is of course like First of all, complete idiosyncrasies and so on But if I can just do that quickly There we go So, and then we can go And we've touched on that Mentioned waste, we mentioned water This is a huge point Spread of AIMAR Because interestingly enough When I was following the research Especially in Southern Africa You can see something here That countries with lower antimicrobials In the global south for example Parts of Southern Africa Has in absolute numbers Lower antimicrobial use Than for example Some parts of Europe and the United States In absolute numbers But we see higher resistant rates here Which is quite interesting So there's more going on than just A linear connection between Amount of use and resistance It's also, as we've talked about How it's used It's not just, it's a quantitative question Not just a quantitative one And it highlights how Health governance, ecological degradation And poor water sanitation Creates what this group talked about Ecological and social environments Right would spread the resistant strains After example So, and based on what I've been reading And reading on this And working And based on the workshops We've been having It's, I think we know Perfectly agree that It's necessary to consider The purpose of any more education In these kind of questions And broaden it Especially around The spread of resistant syndrome In lower immune characteristics And here we have another example Which I found quite interesting We have that magnitude Of environmental reservoirs Such as waste water In which pathogens might be harbored Justifications of climate change Or salinization Of course social justice Inequality And resource addiction And antimicrobial residues We were talking about For example For eggs and milk It seems like the general recommendation Is just seven days And if you build a cow for seven days For a bear farm That's quite a long time And for layers with eggs Seven days of eggs Not being able to sell them That's also quite a lot So, in those instances Toys have increased the danger Of a more aggressive curve So, as Dr. Silvini Usually says to me Is that also Antimicrobial resistance Antimicrobial resistance Is also a genetic question It's a question of genetics Of resistant genes being shared So you can think about The dark side So, it's just I think the crucial thing That I'm taking away From your discussion From listening in Is also around We need to really open up How we think about the Immoral education Of preventing The kind of emergency resistance We need to go broader So, with that said I would like to say Thank you so much For your time Any last comments or reflections Before we close? Otherwise Yes Just in addition To your point She said We need to involve Policies But with the policies I want to understand That as of now There are some policies Which are in place But they are not being followed So, implementation as well Is vital We need to implement What we have formulated As policies And maybe review Those policies After a certain period To see how effective Are these policies To our community Or to our country Definitely So, that's the classic You can say within health Like public health Is kind of an implementation gap We can have a very good policy We have a one health AMR policy for ZIM We have the WHO But how is it in practice? That's also the challenge It's not just having a focus And content We also need to find out How to do things So, with that said Thank you so much again We really appreciate For me and for the meeting I hear that the lunch is ready So we can have something to eat Close

out the day So, again Thank you so much And for your time And your engagement I will be in contact Thank you so much
