# Supplementary material for: Antimicrobials as cornerstones and quick fixes Zimbabwean in healthcare and society: Health practitioners´ critical reflections on two stories of antimicrobial use as part of antimicrobial resistance (AMR) education
Source: PLOS Glob Public Health. 2025 Jul 7;5(7):e0004793. doi: 10.1371/journal.pgph.0004793 (PMC12233256; doi:10.1371/journal.pgph.0004793)
Supplement: Attachment — Submitted filename: Response to Reviewers.docx [file pgph.0004793.s005.docx]

**Response to reviewers**

***Antimicrobials as cornerstones and quick fixes in healthcare and society: health practitioners´ critical reflections on two stories of antimicrobial use as part of antimicrobial resistance (AMR) education***

We greatly appreciate the input from the reviewers and editors in providing valuable feedback on the

paper. My co-author and I have now worked through the paper and addressed the reviewer's comments. Reviewers’ comments and our response and revisions are outlined in the table below.

| **Reviewer #1:** | **Responses** |
| --- | --- |
| ***Conceptual aspects*** |  |
| Define the object of the study and its scope when making arguments, theoretical discussions and conclusions. | We have carefully defined the object of study and its scope throughout the paper when making arguments, theoretical discussions and conclusions. |
| Contextualize/ limit the framework of the research.  For example, the main global causes of morbidity and mortality are non-communicable diseases, while in developing countries, communicable diseases, closely linked to the use of AMR, are predominant. | We have contextualised/ limited the framing of the research. See the opening statement: Antimicrobial resistance (AMR) is a major global public health crisis with developing countries in major crises due to the high burden of infectious disease. In 2021, out of 7.75 million deaths attributed to bacterial infections, 4.71 million suffered from AMR associated conditions such as lower respiratory tract, blood stream or intra-abdominal infections [(IHME, 2024)](https://www.zotero.org/google-docs/?phSLIw). |
| Describe the social and health dynamics of the study area, specifically in the functioning and structure of the health system for the international audience of the journal.  For example, are there primary health care policies? Are there levels of health care? Why is the study carried out in the selected hospitals? These are some of the concerns that arise when trying to understand the social and health framework that runs through the use of AMR and education. | We have described the social and health dynamics of the study areas with focus on functioning and structure of the health system. This includes outlining the levels of health care and why the specific hospitals were chosen. |
| Add a thoughtful debate on the solid focus on the **social determinants of health** and the **“risk factors”** mentioned in the manuscript would enrich the theoretical discussion. | Have added a discussion on **social determinants of health** and the **risk factors** to the paper to enrich the theoretical discussion in both the introduction and discussion sections. |
| ***Methodological aspects:*** |  |
| Specify the methodological approach of the manuscript. Be clear it is a qualitative study.  The objective allows us to assume that it is in an exploratory phase of the analysis, however, the scope of the same (healthcare and society) and the conclusions of the authors exceed this methodological framework. | The methodological approach of the manuscript has been specified and it is  clarified that it is a qualitative study.  We have aligned the objective with the scope and conclusion of the paper.  Read through again and be clear how the discussion and conclusions align with the methodological framework.  Make sure the two stories of antimicrobials are engaged with in discussion and conclusion. |
| This suggestion is valid for the manuscript in general, for example, in the case of the title it is desirable that the authors make reference to the place where the study is carried out. | This has been addressed and specifically the title has been revised to make reference to the place where the study is carried out. |
| In addition, the objectives should mention the place and time frame in which the research takes place. | The objectives and intro text have been revised to mention the place and time frame in which the research takes place. |
| Specify the arguments that justify the study in the defined area and the selected hospitals   - (what population do they serve? - What is their size? - Is there a criterion based on health demand? - Are these health centers with the highest record of AMR prescription?). | We have developed the justifying arguments for the study population and site. This includes outlining how this study was conducted at two main teaching hospitals in the country providing an ideal setting to explore the values and principles of antimicrobial resistance (AMR) both in practice and in the training of future health practitioners. |
| Qualitative studies are interested in obtaining a deep understanding of people's perspectives in relation to phenomena that occur in a specific place and time. This gives them a different scope from quantitative studies.  In this sense, it is contradictory to refer to "health systems and society." | We have clarified that the paper is engaging with Zimbabwean health care in Harare. |
| Briefly describe the method of instrument validation | We have briefly added details on method of instrument validation. |
| Specify how the discourse analysis was carried out. | Details how the analysis of the transcripts was carried out have been added. |
| ***Minor revisions:*** |  |
| Review the general writing of the manuscript to make the text more fluid and avoid redundancies. | The manuscript has been reviewed to increase the flow and avoid redundancies. |
| Consider the length suggested by the journal for the different sections. | While revising the manuscript the length of different sections suggested by the journal has been considered. |

| **Reviewer #2:** | **Responses** |
| --- | --- |
| The introduction lacks clarity about Antimicrobial Resistance (AMR), A proper scientific definition is missing. | The introduction has been rewritten to add clarity on AMR especially around a scientific definition. |
| The methodology section requires a detailed explanation of the Participatory Rural Appraisal (PRA) methods and their relevance to this study. | A detailed explanation of participatory research workshops methods have been added along with their relevance to the study. |
| The demographic profile of the study participants must be included. | We have outlined the demographic profile of the study participants, including how many men and women and how many where doctors, nurses and pharmacists. |
| The study overlooks the impact of cultural and social taboos on health decision-making. | We have added details on the impact of cultural and social taboos on health decision-making. This includes aspects of who makes decisions on when to take medication, communities that don't encourage hospital attendance. We have linked this to health seeking behaviours and barriers to health decision-making |
| The manuscript needs a more extensive literature review to enhance its scientific depth. | We have done a more extensive literature review to enhance its scientific depth. |
| Numerous grammatical and typographical errors, particularly in capitalization, need thorough correction. | A thorough readthrough and correction of grammatical and typographical errors, particularly in capitalization, need has been made. |

| **Reviewer #3:** | **Responses** |
| --- | --- |
| Details on qualitative data analysis and restructuring of quotes/results): | We have addressed the raised details on data analysis and restructuring of quotes/results. |
| ***Abstract***  * line 23 - ‘from’ two Zimbabwean hospitals, not ‘for’ | Changed ‘for’ to ‘from’ |
| ***Introduction***  * line 84 – ‘switch it’ is unclear | Have clarified the sentence. |
| ***Materials and methods*** |  |
| Say a bit about why Zimbabwe, and whether this work is part of/builds on ongoing work in the study area? | Have added details on why Zimbabwe was chosen.  Add that this work is not part of nor builds on ongoing work in the study area. |
| Some justification for the particular sites would be useful and a brief outline of the ongoing work (line 111/112 refer to previous research, it would be useful to briefly outline these studies and their findings). | Added justification for the particular study sites, i.e. teaching hospitals and how they are putting certain values into education around antimicrobials and AMR. Also a brief outline of the ongoing work and previous studies and their findings. |
| Please add a section on how data analysis was undertaken (of transcripts, field notes and written documentation), linking with the participatory method identified – this is important for understanding the results. | Added section on how data analysis was undertaken. |
| line 143-145 sentence appears incomplete, please revise. | Sentence on Line 143-145 has been revised and split into two sentences. |
| would you be able to include details of ‘the two stories’ – how were they developed and how were they used in the workshops (i.e., how were discussions initiated around the images in Figure 1)? | Included details of ´the two stories´, how they were developed and used in the workshops. |
| ***Results*** |  |
| * lines 203-205 – would suggest choosing one quote from the selection that best evidences your point, and include additional details from the quote in the narrative prior to the quote if they are important to include. | We have chosen one quote that best evidences our point and include some additional details from the quote in the narrative prior to the quote. |
| * same as above re. the multiple quotes used in the results. | We reduced the cases of multiple quotes used in the results and selected key quotes to include in the results. |
| ***Discussion***  * could you discuss how you envisage AMR education being enacted, i.e., is this through CPD or curricula training, or community awareness events. Who is delivering/receiving the education? Are there any barriers to delivery of the education proposed? | We have added a discussion on how we envisage AMR education being enacted, referring back to the choice of study sites as the teaching hospitals where AMR education can be institutionalized through the involvement of health practitioners engaging in ongoing preventive efforts with patients. AMR education is a multi-faceted approach that includes diverse strategies such as health practitioners' education through curricula development and training, patient education, and continuous professional development.  Additionally, policy review, particularly through the AMR policy document, plays a crucial role in shaping effective educational strategies. The Zimbabwean One Health National Action Plan on AMR provides a framework for implementing these efforts. However, barriers such as time and resource limitations may hinder delivery. Despite these challenges, framing AMR education as a key preventive effort can significantly reduce the long-term need for antimicrobials. |
| * it would be useful to situate the findings in the context of other education initiatives/models for AMR education in the country/region. | We have situated the findings in the context of other educational initiatives for AMR education in Zimbabwe, focusing on the AMR educational efforts as part of the  the One Health AMR NAP in Zimbabwe. |
| * line 406, delete ‘Collingon’ | Deleted ‘Collingon’ |
| * line 413, should read Ahmed et al. and Coque et al. | Added “et al.” for both references. |
| * line 415, repetition of ‘for’ | Removed repetition. |
| ***Conclusion*** |  |
| line 430-434, long sentence and difficult to follow, consider breaking this up. | The sentence has been restructured for clarity. |
| ***Acknowledgement*** |  |
| line 458, missing closed bracket | Added a closed bracket. |
