# Supplementary material for: Antimicrobials as cornerstones and quick fixes Zimbabwean in healthcare and society: Health practitioners´ critical reflections on two stories of antimicrobial use as part of antimicrobial resistance (AMR) education
Source: PLOS Glob Public Health. 2025 Jul 7;5(7):e0004793. doi: 10.1371/journal.pgph.0004793 (PMC12233256; doi:10.1371/journal.pgph.0004793)
Supplement: Attachment — Submitted filename: Reviewer Comments.docx [file pgph.0004793.s003.docx]

**Reviewer Comments**

The introduction lacks clarity about Antimicrobial Resistance (AMR), and a proper scientific definition is missing.

The methodology section requires a detailed explanation of the Participatory Rural Appraisal (PRA) methods and their relevance to this study.

The demographic profile of the study participants must be included.

The study overlooks the impact of cultural and social taboos on health decision-making.

The manuscript needs a more extensive literature review to enhance its scientific depth.

Numerous grammatical and typographical errors, particularly in capitalization, need thorough correction.
